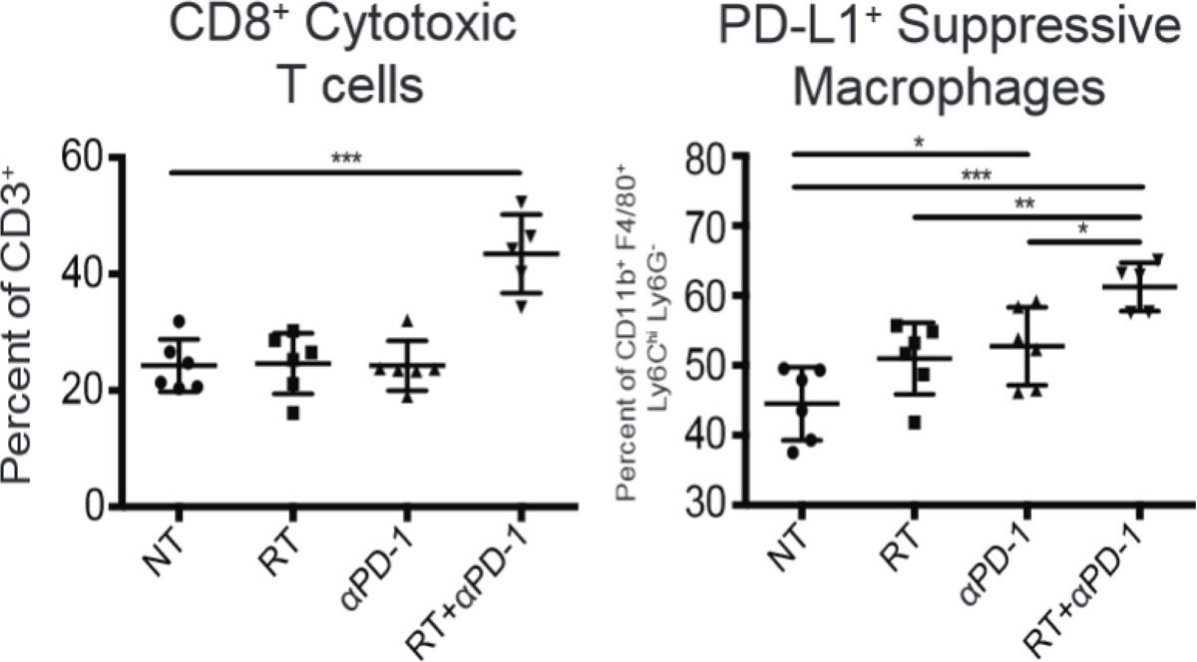# Programmed cell death-1 blockade in combination with stereotactic radiation in an orthotopic mouse model of hepatocellular carcinoma

**DOI:** 10.1186/2051-1426-3-S2-P369

**Published:** 2015-11-04

**Authors:** David Friedman, Jason Baird, Kristina Young, Benjamin Cottam, Zeljka Jutric, Marka Crittenden, Michael Gough, Pippa Newell

**Affiliations:** 1 Earle A. Chiles Research Institute, Providence Cancer Center, Portland, OR, USA; 2 Earle A. Chiles Research Institute, Providence Cancer Center; The Oregon Clinic, Portland, OR, USA; 3 Providence Cancer Center, Portland, OR, USA

## 

Hepatocellular carcinoma (HCC) develops in an environment of chronic inflammation. Curative treatments are plagued by frequent recurrences locally near the treated tumor, regionally within the inflamed liver, and systemically, and yet no adjuvant therapies are known to prevent recurrence. Here the efficacy of immunotherapy with stereotactic radiation (SBRT) in an immune-competent murine model is evaluated. A syngeneic HCC cell line (Hep 55.1c) is injected into the liver of C57BL/6 mice using ultrasound guidance. The tumors are measured and treated using computed tomography and a single dose of intravenous contrast that is taken up by resident liver macrophages allowing visualization of tumors for at least 30 days. Stereotactic radiation is delivered using a Small Animal Radiation Research Platform. Three doses of 250 ug anti-Programmed Cell Death-1 antibody (αPD-1) are delivered concurrently with 30 Gy SBRT in 3 fractions. This combination reduces the growth rate of tumors and improves survival (p < 0.05). Addition of αPD-1 to SBRT is associated with increases in CD8+ cytotoxic T cells based on flow cytometry (Figure 1a) and CD3+ T cells based on six-color immunofluorescence, and this augmentation of immune response is associated with increased ICOS expression on T cells within tumor and spleen. Importantly, the tumors grow out rapidly after αPD-1 is discontinued. The percentage of regulatory T cells (CD4+CD25+FOXP3+) also increases significantly after αPD-1 alone and in combination with SBRT (p < 0.001), suggesting a potential mechanism by which cytotoxic T cells could be rendered anergic. In addition, the combination of αPD-1 plus SBRT induced programmed cell death-1 ligand expression on suppressive macrophages based on flow cytometry (Figure 1b) and immunofluorescence, suggesting another potential mechanism of resistance to long term immune control of the tumors. Conclusion: Tumor response to SBRT can be augmented by concurrent administration of αPD-1 antibody. The efficacy of this combination therapy is transient, however. The environment of chronic immune suppression in most patients affected by HCC adds to the rationale of this immune-radiotherapy combination, but the mechanisms of immune resistance will need to be overcome for this combination to generate lasting immunity.

**Figure 1:**